# Propyl 2-(1*H*-indol-3-yl)acetate

**DOI:** 10.1107/S1600536813027633

**Published:** 2013-10-23

**Authors:** Guo-Min Tang, Wei Xu

**Affiliations:** a Department of Chemical Engineering, Taizhou Institute of Science and Technology, NJUST, Meilan Dong Road No. 8 Taizhou, Taizhou 225300, People’s Republic of China

## Abstract

In the title compound, C_13_H_15_NO_2_, the acetate group [C—C(=O)—O] makes a dihedral angle of 62.35 (13)° with the mean plane of the indole ring system [maximum deviation = 0.011 (3) Å]. In the crystal, mol­ecules are linked by N—H⋯O hydrogen bonds, forming helical chains propagating along [010].

## Related literature

For the use of the title compound as a starting material for the synthesis of platinum complexes with anti­tumor activity, see: Kim *et al.* (1994 ▶). For its use as an inter­mediate in organic synthesis, see: Pandey *et al.* (1997 ▶). For the synthesis of indole-3-acetic acid, see: Johnson & Donald (1973 ▶). For standard bond-length data, see: Allen *et al.* (1987 ▶).
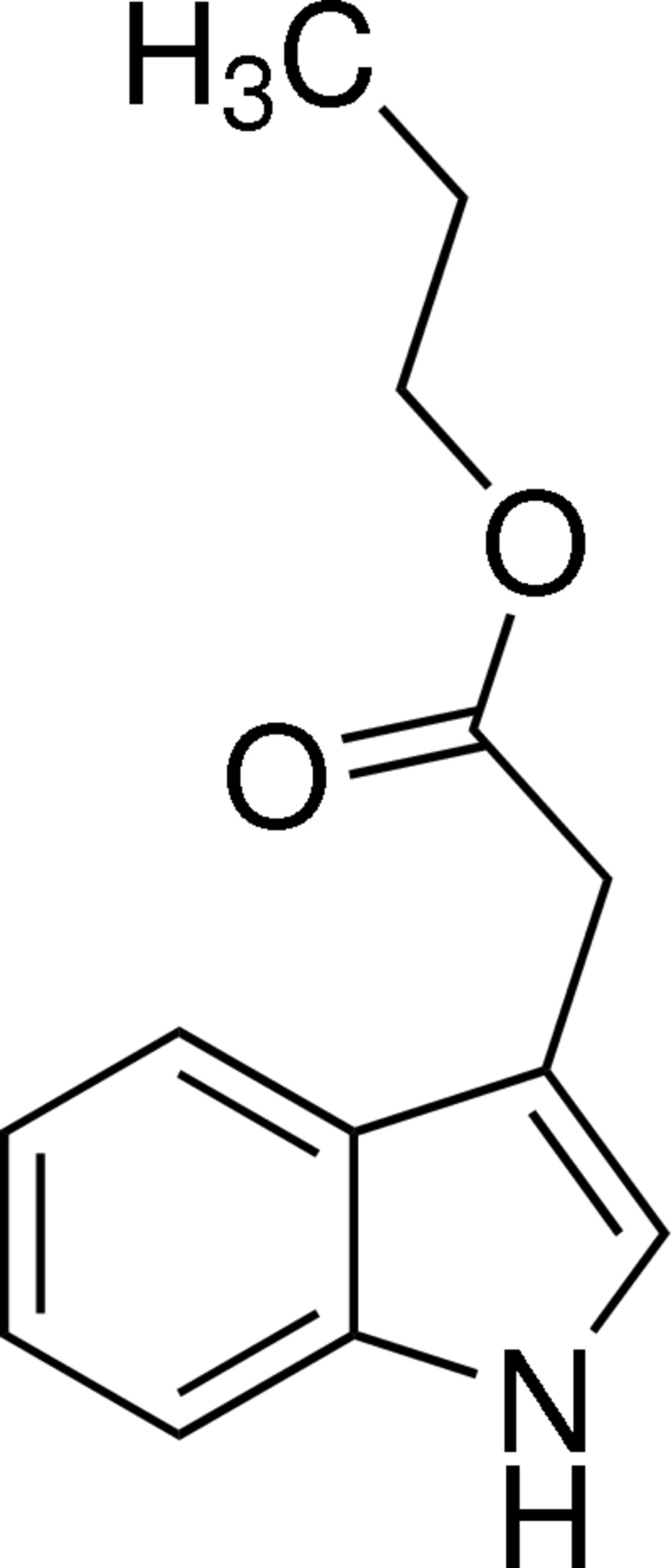


## Experimental

### 

#### Crystal data

C_13_H_15_NO_2_
*M* *_r_* = 217.26
Monoclinic, 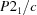

*a* = 7.8230 (16) Å
*b* = 8.1740 (16) Å
*c* = 18.994 (4) Å
β = 97.18 (3)°
*V* = 1205.1 (4) Å^3^
*Z* = 4
Mo *K*α radiation
μ = 0.08 mm^−1^
*T* = 293 K
0.30 × 0.20 × 0.10 mm

#### Data collection

Enraf–Nonius CAD-4 diffractometer
Absorption correction: ψ scan (North *et al.*, 1968 ▶) *T*_min_ = 0.976, *T*_max_ = 0.992
2387 measured reflections
2210 independent reflections
1463 reflections with *I* > 2σ(*I*)
*R*_int_ = 0.083
3 standard reflections every 200 reflections intensity decay: 1%

#### Refinement

*R*[*F* ^2^ > 2σ(*F* ^2^)] = 0.063
*wR*(*F* ^2^) = 0.183
*S* = 1.00
2210 reflections
145 parameters
H-atom parameters constrained
Δρ_max_ = 0.22 e Å^−3^
Δρ_min_ = −0.30 e Å^−3^

### 

Data collection: *CAD-4 Software* (Enraf–Nonius, 1989 ▶); cell refinement: *CAD-4 Software*; data reduction: *XCAD4* (Harms & Wocadlo, 1995 ▶); program(s) used to solve structure: *SHELXS97* (Sheldrick, 2008 ▶); program(s) used to refine structure: *SHELXL97* (Sheldrick, 2008 ▶); molecular graphics: *PLATON* (Spek, 2009 ▶); software used to prepare material for publication: *SHELXL97*.

## Supplementary Material

Crystal structure: contains datablock(s) global, I. DOI: 10.1107/S1600536813027633/su2653sup1.cif

Structure factors: contains datablock(s) I. DOI: 10.1107/S1600536813027633/su2653Isup2.hkl

Supplementary material file. DOI: 10.1107/S1600536813027633/su2653Isup3.cml

Additional supplementary materials:  crystallographic information; 3D view; checkCIF report

## Figures and Tables

**Table 1:** Hydrogen-bond geometry (Å, °)

*D*—H⋯*A*	*D*—H	H⋯*A*	*D*⋯*A*	*D*—H⋯*A*
N1—H1*N*⋯O2^i^	0.86	2.13	2.953 (3)	160

Symmetry code: (i) 
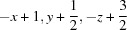
.

**Fig. 1.**
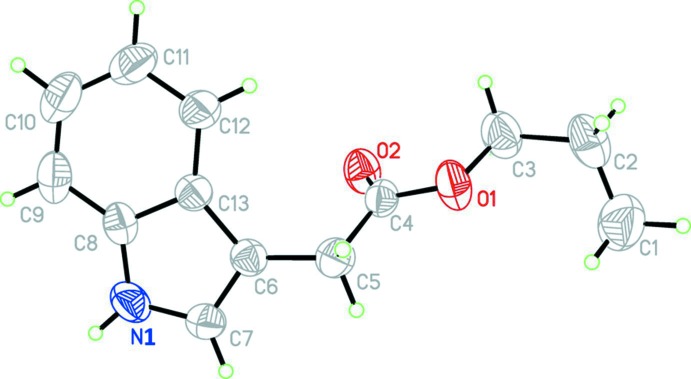
The molecular structure of the title molecule, with atom labelling. The displacement ellipsoids are drawn at the 30% probability level.

**Fig. 2.**
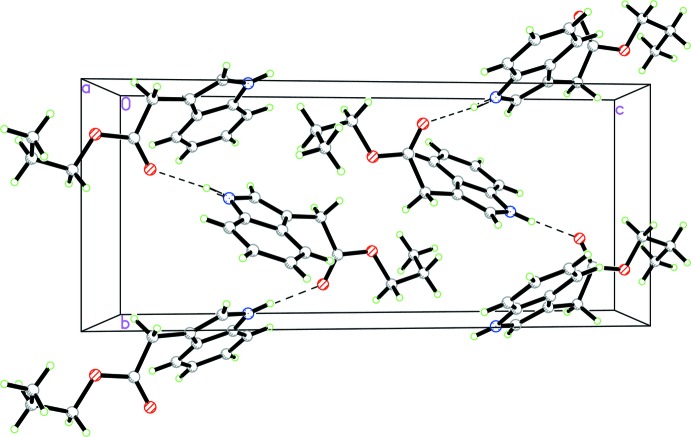
A view along the a axis of the crystal packing of the title compound. The hydrogen bonds are shown as dashed lines (see Table 1 for details).

## supplementary crystallographic information

### 1. Comment

Indole derivatives are some of the most effective anticancer agents currently available. The title compound is a starting material for the synthesis of platinum complexes with antitumor activity (Kim *et al.*, 1994) and is also an important intermediate in organic synthesis (Pandey *et al.*, 1997). As part of our studies of the synthesis and characterization of such compounds, we herein report on the crystal structure of the title compound.

The molecular structure of the title compound is shown in Fig. 1. The bond lengths (Allen *et al.*, 1987) and angles are within normal ranges. The acetate group [C5-C4(═O2)-O1] makes a dihedral angle of 62.35 (13) ° with the mean plane of the indole ring system [N1/C6-C13; maximum deviation = 0.011 (3) Å for atom C9].

In the crystal, molecules are linked by N—H···O hydrogen bonds forming helical chains propagating along the b axis direction (Table 1 and Fig 2).

### 2. Experimental

Indole-3-acetic acid was synthesized following a literature procedure (Johnson & Donald, 1973). The title compound was synthesized by adding indole-3-acetic acid (10 g, 0.057 mol) and 100 mL of dichloromethane to a three-neck flask with stirring and cooled in an ice bath. 4.3 mL of thionyl chloride was added drop wise, after the solution was stirred for a further 10 min. 15 mL of 1-propanol was then added and the reaction was followed using TLC until completion. The title compound was obtained as a light yellow solid [Yield = 10.5 g, 0.048 mol]. Recrystallization with ethanol gave yellow block-like crystals, suitable for X-ray diffraction analysis.

### 3. Refinement

H atoms were positioned geometrically (N-H = 0.86 Å, C—H = 0.93, 0.97 and 0.96 Å for CH, CH_2_ and CH_3_ H atoms, respectively) and refined as riding atoms with *U*_iso_(H) = 1.5*U*_eq_(C-methyl) and = 1.2*U*_eq_(N,C) for other H atoms.

### Crystal data

**Table:**

C_13_H_15_NO_2_	*F*(000) = 464
*M**_r_* = 217.26	*D*_x_ = 1.198 Mg m^−^^3^
Monoclinic, *P*2_1_/*c*	Mo *K*α radiation, λ = 0.71073 Å
Hall symbol: -P 2ybc	Cell parameters from 25 reflections
*a* = 7.8230 (16) Å	θ = 10–13°
*b* = 8.1740 (16) Å	µ = 0.08 mm^−^^1^
*c* = 18.994 (4) Å	*T* = 293 K
β = 97.18 (3)°	Block, yellow
*V* = 1205.1 (4) Å^3^	0.30 × 0.20 × 0.10 mm
*Z* = 4	

### Data collection

**Table:**

Enraf–Nonius CAD-4 diffractometer	1463 reflections with *I* > 2σ(*I*)
Radiation source: fine-focus sealed tube	*R*_int_ = 0.083
Graphite monochromator	θ_max_ = 25.4°, θ_min_ = 2.2°
ω/2θ scans	*h* = 0→9
Absorption correction: ψ scan (North *et al.*, 1968)	*k* = 0→9
*T*_min_ = 0.976, *T*_max_ = 0.992	*l* = −22→22
2387 measured reflections	3 standard reflections every 200 reflections
2210 independent reflections	intensity decay: 1%

### Refinement

**Table:**

Refinement on *F*^2^	Primary atom site location: structure-invariant direct methods
Least-squares matrix: full	Secondary atom site location: difference Fourier map
*R*[*F*^2^ > 2σ(*F*^2^)] = 0.063	Hydrogen site location: inferred from neighbouring sites
*wR*(*F*^2^) = 0.183	H-atom parameters constrained
*S* = 1.00	*w* = 1/[σ^2^(*F*_o_^2^) + (0.1*P*)^2^ + 0.3*P*] where *P* = (*F*_o_^2^ + 2*F*_c_^2^)/3
2210 reflections	(Δ/σ)_max_ < 0.001
145 parameters	Δρ_max_ = 0.22 e Å^−^^3^
0 restraints	Δρ_min_ = −0.30 e Å^−^^3^

### Special details

**Table:**

Geometry. All e.s.d.'s (except the e.s.d. in the dihedral angle between two l.s. planes) are estimated using the full covariance matrix. The cell e.s.d.'s are taken into account individually in the estimation of e.s.d.'s in distances, angles and torsion angles; correlations between e.s.d.'s in cell parameters are only used when they are defined by crystal symmetry. An approximate (isotropic) treatment of cell e.s.d.'s is used for estimating e.s.d.'s involving l.s. planes.
Refinement. Refinement of *F*^2^ against ALL reflections. The weighted *R*-factor *wR* and goodness of fit *S* are based on *F*^2^, conventional *R*-factors *R* are based on *F*, with *F* set to zero for negative *F*^2^. The threshold expression of *F*^2^ > σ(*F*^2^) is used only for calculating *R*-factors(gt) *etc*. and is not relevant to the choice of reflections for refinement. *R*-factors based on *F*^2^ are statistically about twice as large as those based on *F*, and *R*- factors based on ALL data will be even larger.

### Fractional atomic coordinates and isotropic or equivalent isotropic displacement parameters (Å^2^)

**Table:**

	*x*	*y*	*z*	*U*_iso_*/*U*_eq_	
N1	0.4476 (3)	0.5291 (3)	0.76568 (11)	0.0551 (6)	
H1N	0.4531	0.5727	0.8071	0.066*	
O1	0.6827 (3)	0.2944 (2)	0.50091 (9)	0.0572 (6)	
C1	0.9681 (5)	0.2675 (5)	0.4162 (2)	0.0913 (12)	
H1A	1.0120	0.2982	0.3731	0.137*	
H1B	1.0455	0.1909	0.4418	0.137*	
H1C	0.9582	0.3630	0.4448	0.137*	
O2	0.6241 (3)	0.1504 (2)	0.59452 (9)	0.0580 (6)	
C2	0.7950 (5)	0.1904 (4)	0.39885 (15)	0.0677 (9)	
H2A	0.8070	0.0929	0.3707	0.081*	
H2B	0.7205	0.2658	0.3700	0.081*	
C3	0.7106 (5)	0.1444 (4)	0.46239 (16)	0.0684 (9)	
H3A	0.6015	0.0904	0.4478	0.082*	
H3B	0.7838	0.0702	0.4925	0.082*	
C4	0.6405 (3)	0.2803 (3)	0.56652 (12)	0.0417 (6)	
C5	0.6233 (3)	0.4467 (3)	0.59888 (13)	0.0462 (6)	
H5A	0.7379	0.4897	0.6132	0.055*	
H5B	0.5672	0.5189	0.5626	0.055*	
C6	0.5254 (3)	0.4523 (3)	0.66121 (12)	0.0408 (6)	
C7	0.5740 (3)	0.5333 (3)	0.72294 (13)	0.0493 (7)	
H7A	0.6799	0.5847	0.7344	0.059*	
C8	0.3090 (3)	0.4439 (3)	0.73219 (13)	0.0461 (6)	
C9	0.1503 (4)	0.4081 (4)	0.75382 (17)	0.0608 (8)	
H9A	0.1222	0.4447	0.7973	0.073*	
C10	0.0360 (4)	0.3173 (4)	0.7092 (2)	0.0685 (9)	
H10A	−0.0707	0.2901	0.7228	0.082*	
C11	0.0784 (4)	0.2654 (4)	0.64391 (18)	0.0637 (8)	
H11A	−0.0011	0.2038	0.6145	0.076*	
C12	0.2337 (3)	0.3022 (3)	0.62148 (15)	0.0516 (7)	
H12A	0.2590	0.2669	0.5774	0.062*	
C13	0.3536 (3)	0.3938 (3)	0.66602 (12)	0.0401 (6)	

### Atomic displacement parameters (Å^2^)

**Table:**

	*U*^11^	*U*^22^	*U*^33^	*U*^12^	*U*^13^	*U*^23^
N1	0.0693 (15)	0.0572 (14)	0.0401 (11)	−0.0058 (12)	0.0115 (11)	−0.0130 (11)
O1	0.0821 (14)	0.0501 (11)	0.0442 (10)	0.0020 (9)	0.0262 (10)	−0.0014 (8)
C1	0.091 (3)	0.092 (3)	0.099 (3)	−0.007 (2)	0.044 (2)	−0.015 (2)
O2	0.0766 (14)	0.0506 (11)	0.0497 (11)	0.0029 (10)	0.0196 (10)	0.0071 (9)
C2	0.098 (3)	0.0577 (19)	0.0503 (17)	0.0110 (17)	0.0201 (17)	−0.0075 (14)
C3	0.093 (2)	0.0551 (18)	0.0615 (18)	−0.0074 (16)	0.0259 (17)	−0.0124 (15)
C4	0.0379 (13)	0.0513 (15)	0.0365 (12)	0.0005 (11)	0.0068 (10)	0.0026 (11)
C5	0.0498 (14)	0.0459 (14)	0.0435 (14)	−0.0043 (12)	0.0084 (12)	0.0024 (11)
C6	0.0408 (13)	0.0426 (13)	0.0385 (12)	0.0022 (11)	0.0028 (10)	−0.0013 (10)
C7	0.0488 (14)	0.0527 (16)	0.0461 (14)	−0.0088 (12)	0.0048 (12)	−0.0076 (12)
C8	0.0525 (15)	0.0414 (14)	0.0464 (14)	0.0074 (11)	0.0139 (12)	−0.0012 (11)
C9	0.0635 (18)	0.0532 (17)	0.072 (2)	0.0104 (15)	0.0338 (16)	0.0031 (15)
C10	0.0427 (16)	0.0605 (19)	0.105 (3)	0.0053 (14)	0.0206 (17)	0.0063 (18)
C11	0.0389 (15)	0.0631 (19)	0.086 (2)	0.0008 (13)	−0.0035 (14)	−0.0015 (16)
C12	0.0414 (14)	0.0563 (17)	0.0553 (15)	0.0044 (12)	−0.0010 (12)	−0.0069 (13)
C13	0.0388 (13)	0.0414 (13)	0.0390 (13)	0.0027 (10)	0.0012 (10)	0.0004 (11)

### Geometric parameters (Å, º)

**Table:**

N1—C7	1.356 (3)	C5—C6	1.489 (3)
N1—C8	1.375 (3)	C5—H5A	0.9700
N1—H1N	0.8600	C5—H5B	0.9700
O1—C4	1.333 (3)	C6—C7	1.359 (3)
O1—C3	1.458 (3)	C6—C13	1.440 (3)
C1—C2	1.493 (5)	C7—H7A	0.9300
C1—H1A	0.9600	C8—C9	1.387 (4)
C1—H1B	0.9600	C8—C13	1.407 (3)
C1—H1C	0.9600	C9—C10	1.371 (4)
O2—C4	1.201 (3)	C9—H9A	0.9300
C2—C3	1.495 (4)	C10—C11	1.390 (5)
C2—H2A	0.9700	C10—H10A	0.9300
C2—H2B	0.9700	C11—C12	1.370 (4)
C3—H3A	0.9700	C11—H11A	0.9300
C3—H3B	0.9700	C12—C13	1.399 (4)
C4—C5	1.506 (4)	C12—H12A	0.9300
			
C7—N1—C8	109.1 (2)	C6—C5—H5B	108.4
C7—N1—H1N	125.4	C4—C5—H5B	108.4
C8—N1—H1N	125.4	H5A—C5—H5B	107.4
C4—O1—C3	117.8 (2)	C7—C6—C13	105.7 (2)
C2—C1—H1A	109.5	C7—C6—C5	125.8 (2)
C2—C1—H1B	109.5	C13—C6—C5	128.1 (2)
H1A—C1—H1B	109.5	N1—C7—C6	110.9 (2)
C2—C1—H1C	109.5	N1—C7—H7A	124.5
H1A—C1—H1C	109.5	C6—C7—H7A	124.5
H1B—C1—H1C	109.5	N1—C8—C9	130.8 (3)
C1—C2—C3	114.1 (3)	N1—C8—C13	107.0 (2)
C1—C2—H2A	108.7	C9—C8—C13	122.3 (3)
C3—C2—H2A	108.7	C10—C9—C8	118.1 (3)
C1—C2—H2B	108.7	C10—C9—H9A	121.0
C3—C2—H2B	108.7	C8—C9—H9A	121.0
H2A—C2—H2B	107.6	C9—C10—C11	120.4 (3)
O1—C3—C2	107.6 (2)	C9—C10—H10A	119.8
O1—C3—H3A	110.2	C11—C10—H10A	119.8
C2—C3—H3A	110.2	C12—C11—C10	122.0 (3)
O1—C3—H3B	110.2	C12—C11—H11A	119.0
C2—C3—H3B	110.2	C10—C11—H11A	119.0
H3A—C3—H3B	108.5	C11—C12—C13	118.9 (3)
O2—C4—O1	122.9 (2)	C11—C12—H12A	120.6
O2—C4—C5	126.7 (2)	C13—C12—H12A	120.6
O1—C4—C5	110.4 (2)	C12—C13—C8	118.3 (2)
C6—C5—C4	115.7 (2)	C12—C13—C6	134.4 (2)
C6—C5—H5A	108.4	C8—C13—C6	107.3 (2)
C4—C5—H5A	108.4		
			
C4—O1—C3—C2	−166.9 (2)	C13—C8—C9—C10	1.6 (4)
C1—C2—C3—O1	62.5 (4)	C8—C9—C10—C11	−1.0 (4)
C3—O1—C4—O2	−0.4 (4)	C9—C10—C11—C12	0.0 (5)
C3—O1—C4—C5	177.9 (2)	C10—C11—C12—C13	0.5 (4)
O2—C4—C5—C6	−20.2 (4)	C11—C12—C13—C8	0.1 (4)
O1—C4—C5—C6	161.6 (2)	C11—C12—C13—C6	179.0 (3)
C4—C5—C6—C7	134.1 (3)	N1—C8—C13—C12	179.3 (2)
C4—C5—C6—C13	−54.7 (3)	C9—C8—C13—C12	−1.2 (4)
C8—N1—C7—C6	−0.3 (3)	N1—C8—C13—C6	0.1 (3)
C13—C6—C7—N1	0.3 (3)	C9—C8—C13—C6	179.6 (2)
C5—C6—C7—N1	173.2 (2)	C7—C6—C13—C12	−179.3 (3)
C7—N1—C8—C9	−179.4 (3)	C5—C6—C13—C12	8.2 (5)
C7—N1—C8—C13	0.1 (3)	C7—C6—C13—C8	−0.3 (3)
N1—C8—C9—C10	−179.0 (3)	C5—C6—C13—C8	−172.9 (2)

### Hydrogen-bond geometry (Å, º)

**Table:**

*D*—H···*A*	*D*—H	H···*A*	*D*···*A*	*D*—H···*A*
N1—H1*N*···O2^i^	0.86	2.13	2.953 (3)	160

Symmetry code: (i) −*x*+1, *y*+1/2, −*z*+3/2.
